# Thermoplastic elastomer based microfluidic gradient generator for cell culture and drug testing

**DOI:** 10.1007/s10544-026-00816-y

**Published:** 2026-04-25

**Authors:** Kebin Li, Byeong-Ui Moon, Liviu Clime, Ljuboje Lukic, Keith Morton, Lidija Malic, Anu David, Christophe Faure, Teodor Veres

**Affiliations:** 1 National Research Council Canada, Boucherville, Canada; 2 CHU Saint-Justine Research Center, University of Montreal, Montreal, Canada; 3 Centre for Research and Applications in Fluidic Technologies (CRAFT), University of Toronto, Toronto, Canada

**Keywords:** Microfluidic concentration gradient generators, Microfluidic devices, Thermoplastic elastomer, Cell culture, Drug testing

## Abstract

Microfluidic systems are increasingly utilized in preclinical research, however, developing platforms that are compatible with large-scale manufacturing remains a critical challenge. In this regard, we show that thermoplastic elastomer (TPE)-based microfluidic chips could be a viable alternative to traditional poly(dimethylsiloxane) (PDMS) devices for drug testing applications. Microfluidic concentration gradient generators are essential components in these systems, enabling local spatial and temporal control of biochemical stimuli for applications such as cellular response studies and drug screening. TPEs offer key advantages, especially, optical transparency, flexibility, and compatibility with high-throughput fabrication processes compared to PDMS, thereby enhancing commercialization potential. This study demonstrates the design and fabrication of TPE-based microfluidic devices incorporating concentration gradient generators that allow precise control over solute distributions. Gradient formation was validated through numerical modeling and co-flow experiments using fluorescent dyes. The platform supported the co-culture of human lung fibroblast cells and human colon adenocarcinoma cells (HT-29), which exhibited coordinated responses to gradients of fetal bovine serum (FBS) and sepantronimum bromide (YM-155). Performance benchmarks against PDMS chips demonstrated comparable functionality, underscoring the potential of TPE-based devices for future scalable and cost-effective drug testing and cellular assays.

**Supplementary Information:**

The online version contains supplementary material available at 10.1007/s10544-026-00816-y.

## Introduction

Traditional high-throughput screening (HTS) methods are often resource-intensive and time-consuming. Microfluidic systems, however, allow for parallel processing of thousands of samples with minimal reagent usage reducing the costs. Microfluidic platforms have significantly improved HTS processes in drug screening, for instance, the development of droplet-based microfluidics enables the encapsulation of single cells or compounds in picolitre droplets, facilitating rapid and efficient screening (Teh et al. 2008). Microfluidics also play a crucial role in personalized medicine by enabling the testing of drug responses on patient-derived cells allowing for the customization of treatment plans based on individual patient profiles. Microfluidic devices were used to culture patient-specific tumor cells and test their responses to various chemotherapeutic agents (Zhang and Nagrath 2013). They are extensively used for toxicity testing, providing a more accurate assessment of a drug’s safety profile. The ability to create complex tissue models and control the microenvironment precisely makes microfluidics an ideal tool for studying drug-induced cellular toxicity. Valle et al. (2022) reviewed the great advancements that could be achieved in using microfluidic concentration gradient generators (MCGG) for toxicological assessments of various biological organisms demonstrating the advantages of MCGG over other techniques and their suitability for several biological models. However, one of the main challenges still facing microfluidic drug testing is the lack of standards across different platforms leading to the variability in device design and fabrication and hence, inconsistent results. Furthermore, scaling up microfluidic systems for industrial applications remains a significant hurdle therefore, scalable manufacturing processes of such platforms are highly anticipated.

The essential principle behind MCGG involves precise and controllable mixing of different fluid streams of varying solute concentrations within a network of microchannels. Diffusion and convection gradients can be controlled by fine tuning microchannel design and adjusting flow rates of the input fluids. Several innovative designs, each with unique advantages have been developed over the last decades: T-sensor gradient generators utilize a simple T-junction to create gradients perpendicular to the flow direction, making them straightforward to implement (Kamholz et al. 1999), tree-like gradient generators employ a branching network of channels to produce more complex and controlled gradients, suitable for intricate experimental setups, and flow-focusing gradient generators offer a different approach using a central stream flanked by sheath streams, allowing for precise gradient formation through controlled focusing and diffusion. The versatility of these devices allows for the creation of various gradient profiles, including linear (Li et al. 2007), exponential (Friedrich et al. 2012), and more complex patterns(Campbell and Groisman 2007; Dertinger et al. 2001; Lin et al. 2004) tailored to specific experimental needs.

These devices are designed to create stable and reproducible chemical concentration gradients, which are essential for a wide range of scientific and engineering applications. In biological research, particularly in the study of cellular responses to chemical stimuli, they can be used to analyze the chemotactic behavior of neutrophils providing insights into mechanisms of immune responses and spatially controlling stem cell proliferation and differentiation in a regulated microenvironment of chemical morphogens (Benedetto et al. 2014; Cui et al. 2020; Demers et al. 2016; Engel et al. 2025). The ability to generate precise concentration gradients in such small volumes has revolutionized experimental approaches in biological, chemical, and materials science disciplines. By enabling the study of cellular responses, chemical reaction kinetics, and environmental effects in a well controlled manner, microfluidic concentration gradient generators have become indispensable tools in current cell culture research. These systems are considered as core, required elements in organ-on-chip and cell-based screening platforms (Ebadi et al. 2020).

To tackle some of the challenges faced with MCGG’s application in cell-based screening and drug testing, the emphasis is on advancing both the design and manufacturing of MCGGs. Zhang et al. (2020) studied drug release and their potential toxicology applications using MCGGs with adjustable concentration profiles by altering feed flow rate ratios. Hu et al. (2018) demonstrated a straightforward, cost-effective method for designing and fabricating MCGGs with two and three inlets which produced varying concentration gradients at different flow velocities. Yadav et al. (2022) developed MCGG for drug testing using a unique approach of shaping ceramic fluid in a lifted Hele-Shaw cell (Islam and Gandhi 2017; Yadav et al. 2022). Moreover, there is a growing interest in incorporating new materials and manufacturing techniques in MCGG fabrication. Hong et al. (2016) reported a concentration gradient generator based on a paper-based microfluidic device paired with cell culture microarrays for high throughput drug screening, whereas, Heuer et al. (2022) introduced a 3D-printed microfluidic gradient generator made from a biocompatible and heat-resistant polyacrylate material using high-resolution 3D printing for rapid point-of-care antimicrobial susceptibility testing diagnostics. Wang et al. (2023) designed and fabricated an uniquely advanced “Christmas-tree-like” MCGGs from polymethyl methacrylate (PMMA) substrates and double-sided tape, demonstrating uniform flow rates across all outlets, with promising applications for a wide range of cell-based studies.

However, to our best knowledge, all MCGG devices reported thus far were made from PDMS because of its straightforward fabrication process (Valle et al. 2022). PDMS is naturally hydrophobic and possesses a porous structure, offering substantial surface area for small hydrophobic drug molecules or metabolites to interact with and be absorbed into (Carius et al. 2024; Grant et al. 2021; van Meer et al. 2017; Winkler and Herland 2021). Furthermore, PDMS’s incompatibility with organic solvents and vapor permeability can lead to undesirable experimental effects and potential artifacts in biological assays (Mukhopadhyay 2007; Regehr et al. 2009; Sackmann et al. 2014). These attributes present a significant challenge for the commercial use of PDMS-based MCGG devices, particularly in mass production. Materials that merge the benefits of manufacturability with those of typical polymer thermoplastics could significantly enhance the commercialization potential of microfluidic devices and facilitate their adoption for pre-clinical applications (Ingber 2022). TPE are a class of thermoplastic materials that show great potential as an alternative for microfluidic devices and cell culture applications due to their broad spectrum of material properties (Borysiak et al. 2013; Lachaux et al. 2017, 2019). TPEs are optically transparent, soft, flexible, stretchable, and compatible with other plastic materials and manufacturing processes, providing a viable route for commercialization efforts. Some main physical properties of TPE and PDMS are compared in a table (supplementary section, Table S1) in this regard. Previously, our group has showcased TPE-based microfluidic platforms with reversible bonding for various applications, including protein patterning (Brassard et al. 2011), modular droplet formation, (McMillan et al. 2021), and cell patterning (Moon et al. 2021). Additionally, we have developed tissue-engineered micro vessels-on-chip, which can be offered as off-the-shelf injectable tissue-laden entity (Moon et al. 2024). The tunable bonding capability of TPE makes these materials extremely versatile, allowing for both conformal attachment and reversible detachment to other thermoplastic materials. This feature can be beneficial for applications such as post-gene expression analysis following drug testing (Chawla et al. 2022).

Therefore, we created TPE-based microfluidic gradient generation devices and demonstrated their potential as a platform for drug testing. First, we show a microfluidic device design featuring a large cell culture chamber with dimensions of either 2 mm by 2 mm or 2 mm by 4 mm, connected by an array of small, perpendicular microfluidic channels to two main flow channels on each side of the chamber. We also show how the TPE-based microfluidic chips were fabricated and demonstrate the generation of concentration gradients on these chips. In this regard, we used Navier-Stokes equation for the conservation of momentum, the continuity of mass (general laminar flow) together with mass balance equations for transported species under diffusion and convection using COMSOL (version 3.4). The simulation results were validated experimentally in the fabricated devices by fluorescent imaging of co-flowing dyes and also by assessing the response of the human lung fibroblast cells to FBS concentration gradient formed on the chips. Finally, we demonstrated how TPE microfluidic chips were used for culturing human colon adenocarcinoma cells (HT-29) and assess their response to the concentration gradient of sepantronium bromide (YM-155) and benchmark their performance to the PDMS devices.

## Materials and methods

### TPE and PDMS device fabrication

**Fig. 1:**
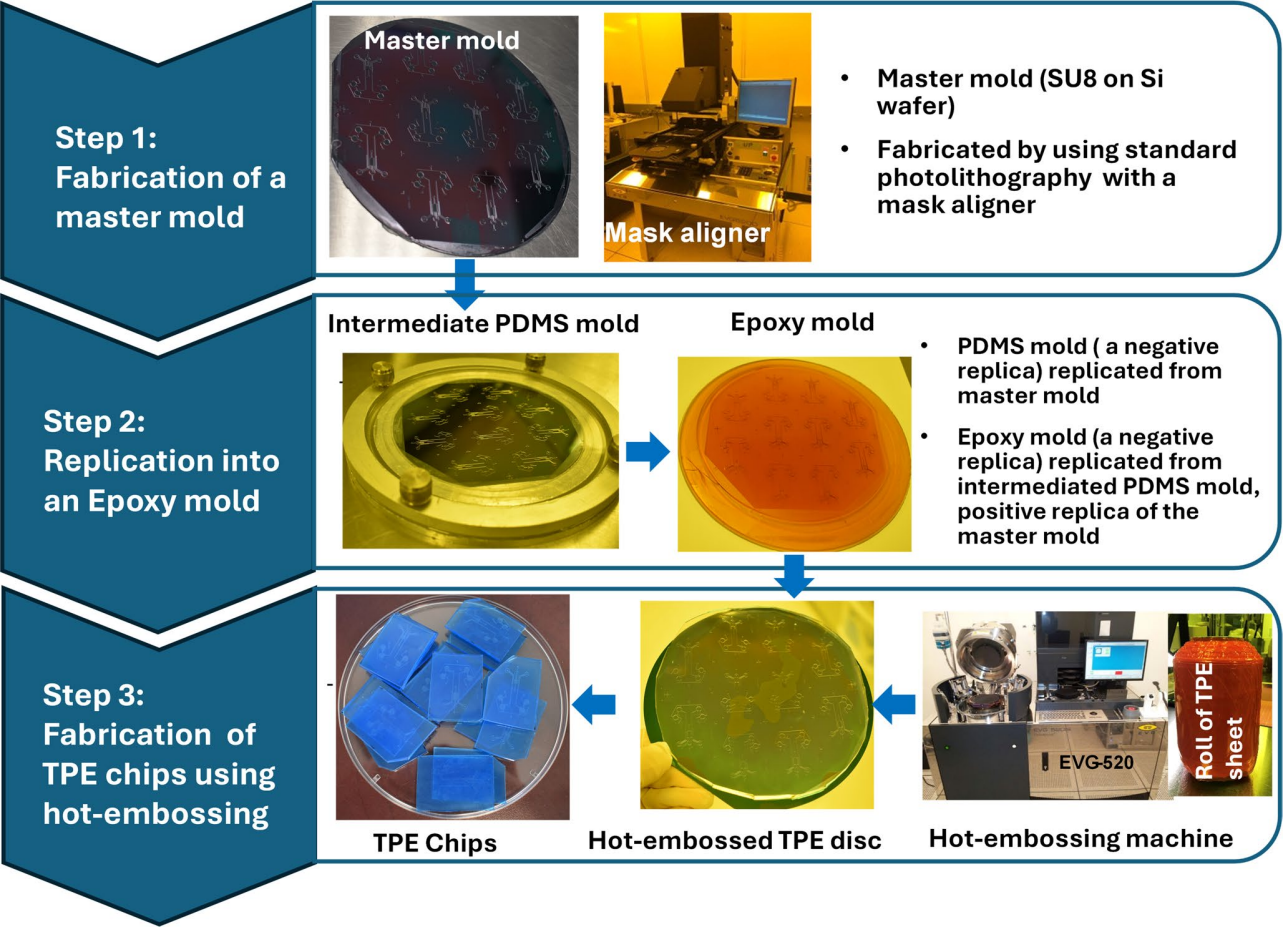
A schematic diagram of process flow chart for TPE chip fabrication by using hot-embossing technique

**Fig. 2:**
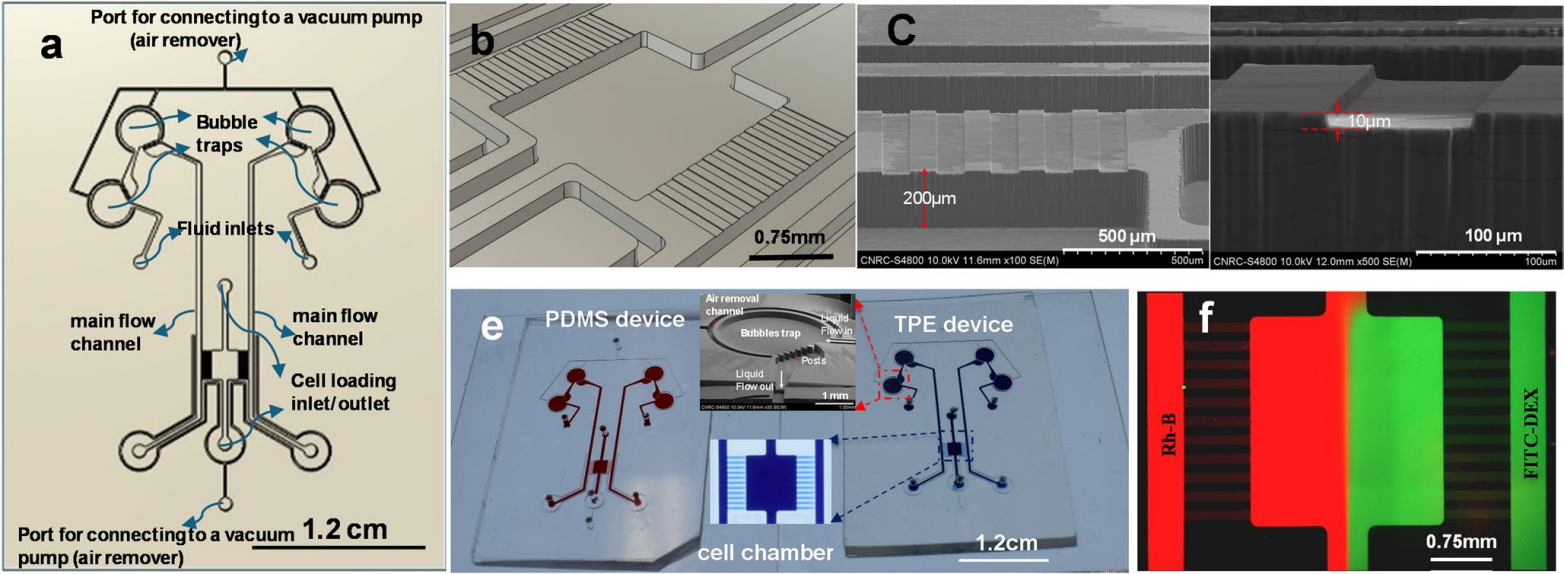
A schematic drawing of the photomask design of the MCGG chip (**a**). Dimensional details of the chamber and fluidic channels are shown in (**b**) with 100 μm wide microchannels. Cross-sectional SEM images of a TPE device with the same width for communication channels (**c** and **d**), indicating that the depth of the cell chamber and the main flow channel is in about 200 μm, while the depth of the communication channels is about 10 μm. (**e**) An image displaying a PDMS device on the left and a TPE device on the right, both bonded on glass slides and filled with red and blue color food dye, respectively. The insets provide a close-up view of a bubble trap (SEM image) and a cell chamber of a TPE chip, which are linked to two main flow channels via an array of communication channels. (**f**) an image of a TPE device undergoing testing with co-flowing Rh-B and FITC-DEX fluorescent dyes (0.2mM) at a flow rate of 1µL/min

Figure 1 depicts a schematic diagram of the fabrication process for TPE chips. It consists of three main steps including the fabrication of a master mold using standard photolithography process, replication of an epoxy mold from the master mold by an intermediated PDMS mold and the fabrication of the TPE chips using hot-embossing. A microfluidic device design featuring a main culturing chamber with dimensions of either 2 mm by 2 mm or 2 mm by 4 mm is shown in Fig. 2 (a). These chambers are connected to two main flow channels on each side through an array of microfluidic channels, enabling the generation of stable concentration gradients within the main chamber. This setup is used to study how cells, such as lung fibroblast cells or human colonic adenocarcinoma cells, respond to changes in their microenvironment. To minimize the adverse effects of flow perturbations from trapped air bubbles or geometric variations during fabrication, the width and the depth of the communication channels are designed to be significantly smaller than those of the main flow channels and the cell chamber. We utilized a two-layered device design, with the communication channel depth targeted at 10 μm, while the cell chamber and the main flow channels are approximately 200 μm deep.

The depth of the communication channels was determined by the thickness of the SU-8 photoresists used in the standard photolithography process for creating a master mold. Initially, a layer of SU-8 photoresist (Gersteltec, Pully, Switzerland) aimed at 10 μm was spin-coated onto a 6-inch silicon wafer (Silicon Quest International, Santa Clara, CA), then pre-baked and exposed to UV light at 365 nm (EVG 620 Mask Aligner, Austria) through a photomask designed with the communication channels. Two photomasks were crafted using computer-aided design (CAD) software (AutoCAD 2022, Autodesk, Inc., Dan Rafael, CA) and printed onto high-resolution transparency sheets (Fineline Imaging, Colorado Springs, CO). After UV exposure, the SU-8 photoresist underwent post-baking and was subsequently developed in propylene glycol monomethyl ether acetate (PGMEA; Sigma-Aldrich, Oakville, ON). The patterned resist microstructures were then rinsed with PGMEA and isopropanol, and the wafer was dried using a stream of nitrogen gas. A second layer of SU-8 photoresist, targeting a thickness of 200 μm, was applied to the silicon wafer patterned with the communication channels, following the same photo-lithography process. The silicon master mold, patterned with two levels of SU-8 photoresist (10 μm for the communication channels and 200 μm for other features like main flow channels, cell chambers, and bubble traps), was eventually hard baked at 135 °C for 2 h on a programmable hot plate (HS40A; Torrey Pines Scientific).

An epoxy mold was fabricated from the SU-8/silicon master using an intermediate PDMS replica (Sylgard 184; Dow Corning, Midland, MI). Liquid PDMS prepolymers, in a10:1 weight ratio of elastomer base to curing agent, were poured onto the master mold and cured at 80 °C for 4 h. The PDMS replica was separated from the master mold and then served as a template to produce an epoxy mold for the TPE hot embossing process. The epoxy mold was made by pouring a mixture of Conapoxy FR-1080 resin (part A) and hardener (part B) (ELANTAS PDG Inc., St. Louis, MO) in a 75:60 weight ratio into a PDMS intermediated mold replicated from the silicone master mold, then thermally cured for 8 h at 80 °C in an oven, following degassing in a vacuum chamber. After the PDMS intermediate template was peeled off the cured epoxy, the epoxy mold underwent a hard bake for 3 h at 180 °C.

TPE, initially received as pellets (Mediprene OF 400 M, Åmål, Sweden), was extruded at 165 °C to create sheets approximately 2 mm thick. These TPE sheets were then hot-embossed using the epoxy mold (EVG^®^ 520HE, EV Group, Schärding, Austria) to form device microstructures. The typical hot embossing was done by applying a force of 5000 N at a temperature of 150 °C for 5 min. The embossing duration was slightly adjusted to ensure the final thickness of the TPE device ranging from 3 mm to 3.5 mm. Subsequently, the hot-embossed TPE substrates were cut into individual chips using a paper cutter (DAHLE 564 Guillotine) guided by the marks embossed on the TPE substrate initially defined in the mask. Access holes for the inlets and outlets were punched into the TPE chips with 1.5 mm diameter biopsy punches (Integra Miltex, Inc., Rietheim-Weilheim, Germany). The TPE chips were assembled by simply pressing the TPE slab onto a 25 mm by 25 mm glass slide (A6729, P211237, KF), ensuring the removal of air bubbles and then subjected to heat treatment at 60 °C for 2 h to strengthen the bond(Moon et al. 2024).

The same PDMS liquid prepolymers, in a 10:1 weight ratio of elastomer base to curing agent, were poured onto the silicon master mold and cured at 80 °C for 4 h. After curing, the PDMS replica was removed from the master mold and cut into individual chips by using a paper cutter (DAHLE 564 Guillotine). Both the PDMS chips and glass slides, measuring 25 mm by 25 mm, were carefully cleaned and rinsed with ethanol, and dried with a stream of air before assembly. PDMS chips were permanently bonded on the glass slides after the surfaces of both the glass slide and the PDMS chip (side with microstructures) were activated by oxygen plasma for 30 s at RF power of 30 W and O_2_ pressure of 500 mTorr (Harrick Plasma Cleaner, Harrick Plasma Inc., Scientific equipment supplier in Ithaca, New York, USA), followed by heat treatment for 5 min at 125 °C. Access holes for the inlets and outlets were created in the PDMS chips using 1.0 mm diameter biopsy punches (Integra Miltex, Inc., Rietheim-Weilheim, Germany).

### SEM characterization

Both PDMS and TPE chips were sliced with a blade across the cell chamber in y-direction for scanning electron microscopy (SEM) characterization using a Hitachi S-4800 electron microscope. To prevent charging, the cross-section surface of the cell chamber was coated with a 10 nm thick platinum thin film using a Leica EM ACE 600 (Leica, UK). The sample was mounted on a sample holder at an approximate angle of 70 degrees. SEM images were captured with accelerating voltages set to 10 keV.

### Flow testing on chips

For flow testing, metal tubes approximately 1 cm long and 1.0 mm (OD) in diameter were inserted into the inlets of the main flow channel of the devices. The opposite end of these pins was connected to a syringe (BD, 1mL or 3 mL) via sterilized silastic tubing with ID of 0.79 mm and OD of 2.39 mm (TYGON, ND-100-65). The two outlets of the flow channels were blocked using homemade stoppers made from 1 cm long pipette tips filled with PDMS resin and cured at 80 °C for 4 h to block the tips. All channels and the cell chamber of the device were primed with 70% ethanol to prevent any initial air bubbles from being trapped inside the chamber and channels, followed by rinsing with DI water. Care was taken to avoid air bubble formation during the washing process. For PDMS chips, any accidentally formed air bubbles inside the cell chamber could be removed by applying negative pressure through a designated bubble removal inlet of in-plane bubble removal channels as indicated in Fig. 2a. The flow rate was controlled with a commercial syringe pump (Harvard Apparatus 11 Elite Programmable Syringe Pump).

Two fluorescent solutions, each with a concentration of 0.2 mM, were prepared by dissolving Rh-B isothiocyanate Dextran (MW = 10,00, Millipore Sigma) and FITC-Dextran (MW = 4,000, Millipore Sigma) with emission wavelengths at respectively 574 nm and 520 nm in DI water. Both Rh-B and FITC-Dextran solutions were utilized in a flow test to validate the concentration gradient on chips. Fluorescent images were acquired using an EVOS FL inverted fluorescence microscope (Thermo Fisher Scientific) equipped with 4× and 10× objectives. The acquired images were subsequently analyzed using ImageJ software (NIH, Bethesda, MD) to quantify the concentration of gradient profiles across the chamber.

### Cell culture

RFP-expressing human lung fibroblasts (RFP-FBs) (RFP-HLF, Angio-Proteomie, Boston, MA) and HT-29 colorectal adenocarcinoma cell line (HTB-38™, ATCC, Manassas, VA) were cultured in a T-25 flask using Dulbecco’s modified Eagle medium (DMEM) with 10% FBS, penicillin (100 units/mL) and streptomycin (100 µg/mL). RFP-FBs and HT-29 cells were used at passage numbers of P6–P8 and P6–P10, respectively. All cells were maintained at 37 °C in a humidified incubator with 5% CO_2_.

### Microfluidic concentration generator cell seeding and culture

Prior to cell seeding into the MCGG chamber, the microfluidic devices were cleaned with 70% ethanol followed by rinsing with DI water and primed with culture media thereafter. For PDMS-glass bonded devices, the assembled chips were cleaned by injecting 70% ethanol and DI water subsequentially through the inlet and outlet ports using a pipette tip, repeated three times. For PDMS-glass bonded devices, any trapped air bubbles were removed using in plane bubble channels by applying negative pressure through a designated bubble removal inlet. For TPE–glass devices, 70% ethanol was used for priming, followed by rinsing with culture media to ensure wettability. Since air is always dissolved within the medium and the medium doped with YM-155 drug, some air bubbles were inevitably trapped inside a syringe and tubing. In addition to two-stage bubble traps integrated into the main flow channels on chip, a microfluidic bubble trap (Peek Bubble trap, Elveflow, France) was incorporated inline in the tubing prior to the device inlet to prevent bubble entering the cell chamber during cell culture.

Cultured cells were detached from the T-25 flask substrate using 0.25% Tryspin-EDTA (Sigma-Aldrich) and counted using an automated cell counter (Countess Ⅱ, Thermo Fisher Scientific, Waltham, MA). FB and HT-29 were used at approximate concentrations, 1 × 10^6^ mL^− 1^ and 5 × 10^6^ mL^− 1^, respectively. Depending on the MCGG chamber size, either 4 µL (2 × 2 mm) or 6 µL (2 × 4 mm) of the suspended cell solution were gently introduced through the inlet port while the outlet was blocked at the end of the seeding step to stop the media flow. Homogeneous cell distributions were confirmed under an inverted microscope EVOS FL Auto Cell Imaging System (Life Technologies, Carlsbad, CA). Following seeding, 1 mL of culture media was added to both inlet and outlet to connect them fluidically. The cells were then cultured in the incubator for 2 days.

### Drug testing

To determine the IC₅₀ value for HT-29 cells, HT-29 cells were cultured in 96-well plates by seeding approximately 15,000 cells per well and culturing them for 1 day. Serial dilutions of sepantronium bromide (YM-155; Selleck Chemicals LLC, Houston, TX) were prepared at concentrations of 1 µM, 5 µM, 10 µM, 30 µM, 60 µM and 100 µM. Following drug exposure, the cells were cultured for additional 2 days.

For drug testing experiments on MCGG devices, a 10 µM YM-155 solution was prepared from a 10 mM stock solution and used to evaluate its effect on cells. The prepared solution was loaded into a disposable syringe and connected to one of the inlets of the MCGG device via fluidic tubing (0.25 mm I.D., 1.0 mm O.D.; IDEX Health & Science, Oak Harbor, WA). The flow rate was controlled using a syringe pump set to a constant flow rate of 0.5 µL/min, while the opposite inlet was simultaneously perfused with culture media at the same flow rate. Drug exposure to the cell chamber was maintained for 2 days.

### Cell viability assay, imaging and analysis

To generate and evaluate a live cell concentration gradient on the MCGG device, cell cytotoxicity was assessed using the LIVE/DEAD Cell Imaging Kit (Thermo Fisher Scientific, Waltham, MA) following 2 days of drug exposure. A staining solution containing the LIVE/DEAD reagents and Hoechst 33,342 (Thermo Fisher Scientific) was prepared at a 1:500 dilution ratio and introduced into the device chamber. Cells were incubated with the staining solution for 20 min at room temperature.

Stained cells were imaged using an EVOS FL inverted fluorescence microscope (Thermo Fisher Scientific) with 4× and 10× objectives. The acquired images were analyzed using ImageJ software (NIH, Bethesda, MD) to quantify the live cell distribution across the chamber. Cell viability was assessed by calculating the ratio of live cells (green fluorescence) to the total number of cells, as determined by Hoechst nuclear staining (blue fluorescence).

To spatially evaluate the gradient, regions of interest (ROIs) measuring 200 μm × 200 μm were cropped at multiple locations across the chamber. At least three distinct regions per chamber were analyzed to ensure representative sampling. The analyzed data were presented as mean ± standard deviation (SD).

### Numerical simulation

To evaluate the fluid behavior of the microfluidic system, a Multiphysics computational fluid dynamics simulation was conducted by using COMSOL (version 3.4). Concentration and concentration gradient field were computed by coupling the incompressible Navier-Stokes (NS) equations, which address the conservation of momentum and mass continuity (general laminar flow), with mass balance equations for species transported through diffusion and convention. The incompressible NS equations are solved through a transient analysis simulation in 3D as implemented in the MEMS/Microfluidic module (Supplementary Information: Incompressible NS Equations).

The computational domain was meshed and adapted to both physics and numerical requirements, utilizing unstructured meshes comprising up to 400,000 elements. Various mesh elements and grid configurations (most of them are tetrahedral meshing filled the simulation domains) were chosen to prevent numerical instabilities and achieve the desired level of detail. The Finite Element Method (FEM) approach facilitated the calculation of the local velocity field once initial and boundary conditions (Supplementary Information: Initial and Boundary Conditions) were applied. The concentration values are then solved through using the mass transport equation as implemented in the Chemical Engineering Module (Supplementary Information: Mass transport Equation) with the velocity field coupled and transferred from solutions of the impressible NS equation.

## Results and discussion

### Device design and fabrication

A schematic of the MCGG chip design is shown in Fig. 2 (a). The chip features a main chamber designed for cell culture, two primary flow channels, and an array of fluidic communication microchannels that connect the main flow channels to the cell culture chamber. An air remover mechanism and bubble traps(Johnson et al. 2009; Lochovsky et al. 2012) are incorporated into the design; the air remover mechanism is applicable only for comparative PDMS devices due to its intrinsic permeability. Figure 2(b) details the dimensions of the chamber and fluidic channels. In one design example, the cell chamber measures 2 mm in width and 2 mm in length, while in the second design, it is 2 mm wide and 4 mm long. The target depth for the cell chamber and main flow channels is 200 μm, while the target depth of the communication channels is 10 μm. In this specific example, each of the communication microchannels are designed to be 100 μm wide and arrayed on a 200 μm pitch. Figure 2(c) shows a SEM image of a fabricated TPE device with a cell chamber dimension of 2 mm wide and approximately 2.2 mm long, and 200 μm deep. The communication channels are confirmed by SEM characterization to be about 10 μm deep (Fig. 2(d). The depth of the cell chamber/main flow channels and communication channels was determined by the SU8 resist thickness used in the photolithography process. Figure 2 (e) shows images of PDMS chip permanently bonded to a glass slide and a TPE device reversibly bonded to a glass slide. For better visualization, the channels and the cell chamber were filled with red and blue colored food dyes dissolved in water. Insets in Fig. 2(e) provide close-up views of a bubble trap designed for the PDMS device and a segment of the cell chamber linked to the main flow channels through an array of communication channels of the TPE device. Figure 2(f) is a fluorescent image of a TPE device tested with co-flowing Rh-B and FITC- Dextran (FITC-DEX) fluorescence probes, at a concentration of approximately 0.2 mM and a syringe-pump controlled flow rate of 1 µL/min.

### Numerical simulations of concentration gradient generator

**Fig. 3:**
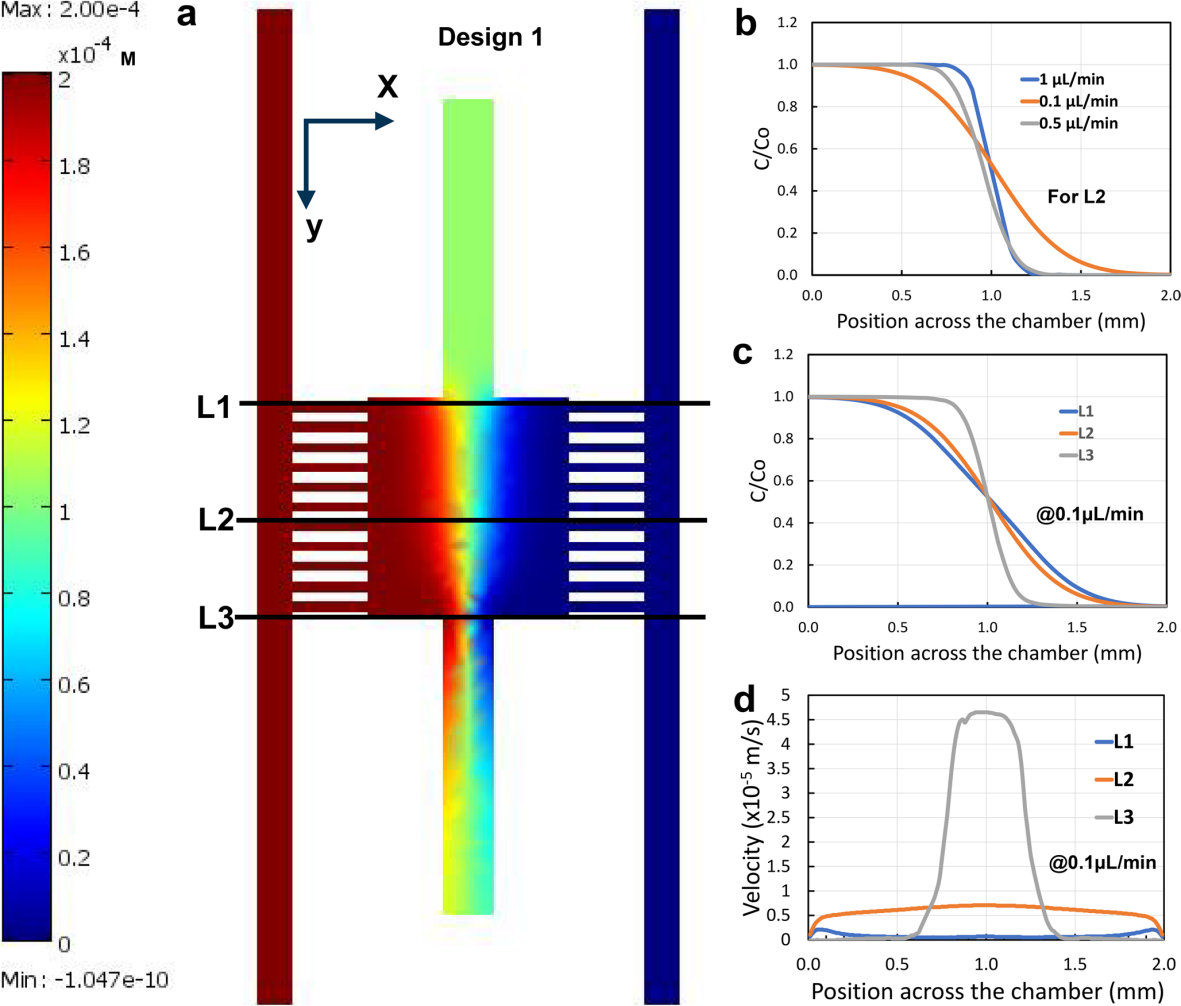
Counter fill plots of the concentration field and profiles of the concentration gradients along the cell chamber for a MCGGs chip, simulated using COMSOL Multiphysics at a flow rate of 0.1µL/min (**a**). A plot showing the normalized concentration across the cell chamber in the x-direction at three different flow rates: 0.1 µL/min, 0.5 µL/min, and 1 µL/min, respectively (**b**), and at three different positions (y) at a flow rate of 0.1 µL/min (**c**). Additionally, a plot of the velocity in the y-direction across the cell chamber (in the x-direction) at three different positions at a flow rate of 0.1µL/min for a chip with the design version 1 (**d**)

Figure 3 demonstrates the simulation results obtained using the chip design 1, with the width of the communication microchannels set to 100 μm, as shown in Fig. 2(c) and (d). The impedance of the two main flow channels had been simplified by removing the air bubble traps from consideration. This did not significantly impact the flow behavior of the main channels, given the constant flow rate supplied by a syringe pump. In this simulation step, the left main flow channel was infused with Rh-B fluorescent dye dissolved in deionized (DI) water at a concentration of 0.2 mM, while the right channel contained pure DI water. Both ends of the main flow channels were closed, allowing the liquid streams to enter the cell chamber via the communication microchannels and exit through air releasing port at the bottom of the chamber, keeping the top air release port closed. The concentration contour fill plots shown in Fig. 3(a) illustrate a well-established concentration gradient of the Rh-B fluorescent dyes across the cell chamber along the X direction, resulting from the diffusion and convection flow of the dyes within the chamber.

Figure 3(c) shows concentration profiles in the cell chamber along different paths (L1, L2, and L3) when the flow rate is set at 0.1 µL/min. At positions L1 and L2, the transition of Rh-B concentration is broad and nearly linear between 0.3 mm < x<1.7 mm. In contrast, at the position L3, the transition is relatively steeper, likely due to the dominance of convection flow in this region, which aligns with the velocity profile shown in Fig. 3(d). The concentration profile across the cell chamber depends also on the flow rate applied in the main flow channel. As illustrated in Fig. 3(b), when the flow rate is increased from 0.1 µL/min to 0.5 µL/min and then to 1 µL/min at the position L2 (the middle of the cell chamber), the concentration gradient of Rh-B along the X direction becomes sharper. This phenomenon can be attributed to the increased convection flow in the cell chamber as the flow rate rises. The linearity of the concentration gradient would further improve when the diffusion coefficient of the dye is increased.

The concentration gradient profile can be further refined by altering the chip design. As shown in the supplementary Figure S1 (a and b), by changing the geometry of the array of the communication microchannels, specifically through stepwise reduction of the microchannel width from 200 μm down to 10 μm. By forgoing uniform microchannel widths, the concentration profile itself becomes more uniform in the main portion of the cell chamber (which means that the linearity of the concentration gradient near the position L3 is getting enhanced too). This can be explained by the increased flow impedance in the narrowest communication microchannels which reduces the convection flow effect near L3. Conversely if the flow direction is reversed, from the narrower to the wider channels, the uniformity of the concentration profile within the cell chamber diminishes. At the position L1, the linearity of the concentration gradient becomes broader, while the transition at L2 and L3 becomes sharper. Alternatively, the concentration gradient profile can be tuned by changing the flow configuration in the setup without changing the chip design. Instead of allowing the liquid streams to exit through the air releasing port at the bottom of the chamber, the liquid stream can exit through the air release port at the top of the chamber too. This means that liquid streams enter the cell chamber via the communication channels and exit through both inlet/outlet ports of the chamber. As shown in supplementary Figure S1 (c), the linearity of the concentration gradient is improved in most of the cell chamber area except for those small regions close to the liquid exiting ports.

To summarize, the concentration gradient profile within the cell chamber can be adjusted by - altering the device design, - adjusting the width and depth of the communication microchannel geometry, and - the dimensions of the cell chamber. Furthermore, the flow direction and the flow rate can also be modified to influence the gradient profile and allowing the flow of media to exit simultaneously from the top and bottom air release ports of the cell chamber.

### Validation of concentration gradient by co-flow of fluorescent dyes on chip

**Fig. 4:**
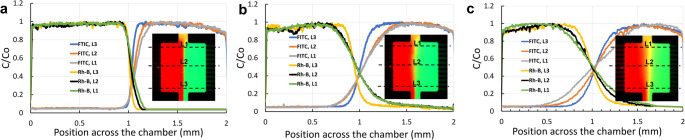
Profiles of concentration gradients for both Rh-B and FITC-Dextran dyes at different positions within the chamber. (**a**) at a flow rate of 1 µL/min, (**b**) at a flow rate of 0.3 µL/min, and (**c**) at a flow rate of 50 nL/min on a PDMS device with design version 1

To validate the concentration gradient profiles modeled using COMSOL simulations, we fabricated MCGG chips using both PDMS and TPE materials. We characterized the concentration gradients on these chips using fluorescent dyes, specifically Rh-B isothiocyanate Dextran and FITC-Dextran, which were dissolved in DI water at a concentration of 0.2 mM. The flow rate was controlled by a syringe pump. The device concentration gradient profiles were characterized by fluorescent images, during co-flow of the two fluorescent dyes. Both devices showed good gradient profiles aligning with the simulation results. At a higher flow rate of 1 µL/min, as illustrated in Fig. 4(a), the transition of the concentration gradient was notably sharper. The concentration gradient profiles for both fluorescent dyes were quite similar; the slight difference might be attributed to their varying molecular weights and consequently, small differences in diffusion coefficients: around 4.0 × 10^–10^ m^2^/s for Rh-B and 2.0 × 10^–10^ m^2^/s for FITC-Dextran (4 kDa) (Gendron et al. 2008; Peng et al. 2021). As the flow rate decreased to 0.3 µL/min, the diffusion portion at the interface between the two flow streams inside the cell chamber increased, causing the transition region of the concentration gradient for both Rh-B and FITC-DEX to widen. Nevertheless, the transition was still relatively sharper at position L3 compared to positions L1 and L2. When the flow rate was further reduced to 50 nL/min, the transition region of the concentration for both dyes became even wider (0.4 mm < x<1.6 mm).

We also analyzed the concentration gradient obtained using microfluidic devices having communication microchannels in the range of 10 μm to 200 μm (design 2). The experimental results illustrated in Figure S2 closely align with the modeling results. When the flow rate is set at 1 µL/min, the transition of the concentration gradient across the cell chamber remains relatively sharp. However, as the flow rate decreases to 100 nL/min and further to 50 nL/min, the transition of the concentration gradient broadens, particularly at the 50 nL/min flow rate. When the liquid streams within the cell chamber moves from regions connected to the narrower communication channels to those connected to wider channels, the concentration transition region for both dyes widen (0.3 mm < x<1.7 mm) compared to the chips with uniform communication channel widths (design 1).

### Effects of drug testing on cells in PDMS and TPE MCGGs

Prior to employing MCGGs for drug testing, we cultured fibroblasts to evaluate the feasibility of the platform to support cell growth. Therefore, RFP-expressed lung fibroblasts were cultured in a PDMS chip under co-flow conditions as a benchmarking step. Culture media supplemented with FBS flowed through one side and FBS-free media flowed though the other side. After 2 days of exposure, we observed an increase in cell numbers in the region exposed to FBS-containing media, whereas the number of cells in the FBS-free region remained relatively constant (See supplementary Information Figure S3).

To assess the cytotoxic effects on HT-29 cells, we selected the small molecule YM-155, an inhibitor of the anti-apoptotic protein Survivin, which has shown induction of cellular death in HT-29 spheroids (Thakuri et al. 2016). Prior to testing the HT-29 cellular response to YM-155 in a MCGGs device, we first established the appropriate working concentration of YM-155 to use on-chip. HT-29 cells were cultured in 96-well culture plates for 1-day followed by exposure to YM-155 at six different concentrations for additional 2 days. Based on the results from the live/dead assay, we observed a significant increase in cell death at concentrations above 5 µM. At 60 µM, more than 90% cells were dead (Supplementary Information Figure S4). The dose-response curve yielded an IC₅₀ of approximately 12.7 µM for 2 days of culture conditions (Supplementary Information Figure S5). This IC₅₀ value was used to select a concentration of approximately 10 µM for YM-155 in all subsequent concentration gradient generation experiments on the devices.

**Fig. 5:**
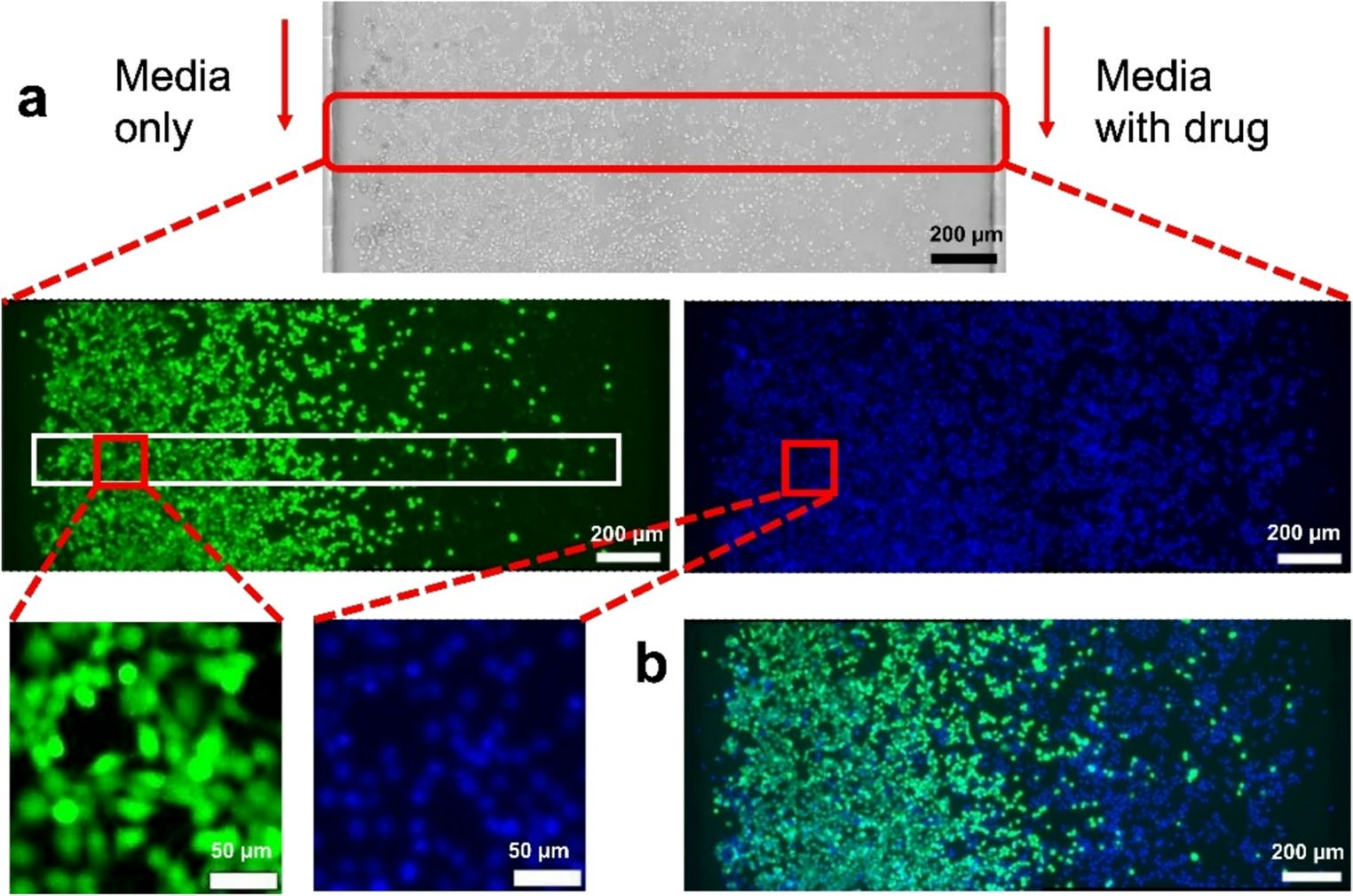
Cytotoxic effects on HT-29 cells cultured on the MCGG in TPE. (**a**) Bright-field image of cultured cells after 2 days of drug exposure. Cells were exposed to co-flowing streams of culture media without drug (left channel) and with drug (right channel). Zoomed-in images show live cell staining and Hoechst-stained nuclei. Cell counts were performed within regions of interest (200 μm × 200 μm) across the chamber, as indicated by the white rectangular outlines. (**b**) Merged image of live cell staining and Hoechst-stained nuclei

For this comparative study, we again used two types of microfluidic devices: PDMS-glass bonded and TPE-glass bonded devices. Although PDMS is a commonly used material for microfluidic systems, its tendency to absorb small molecules is a well-known limitation for drug screening applications (Carius et al. 2024; Grant et al. 2021; van Meer et al. 2017; Winkler and Herland 2021). Additionally, PDMS’s inherent higher material costs, fabrication by casting and vacuum process plasma bonding, present challenges for large-scale manufacturing. In contrast, TPE, a subclass of thermoplastics offers better advantages for both, drug compatibility (low absorption) and direct manufacturability. These advantages make TPE to be a more suitable material for wide scale cell culture and drug testing applications. Figure 5 shows the resulting images of the MCGG device in TPE after two days of co-flowing media and drug solutions. The live cell staining images clearly indicate a cell density gradient along the chamber, in contrast to the control experiment (Supplementary Information Figure S6), while the Hoechst-stained image shows a more uniformly distributed cell population across the chamber. We observed that as the MCGG system operates under continuous flow conditions during drug exposure, some cells undergo apoptosis or detach from the substrate and are subsequently washed away through the outlets. This dynamic loss leads to a reduced cell population and lower density, particularly in the drug-exposed region. To accurately assess the effective percentage of live cells despite this loss, Hoechst staining was used to determine the total cell count, enabling reliable quantification of cellular viability. Each cropped image was analyzed by counting the number of live cells (green) relative to the number of Hoechst-stained cells (blue).

**Fig. 6:**
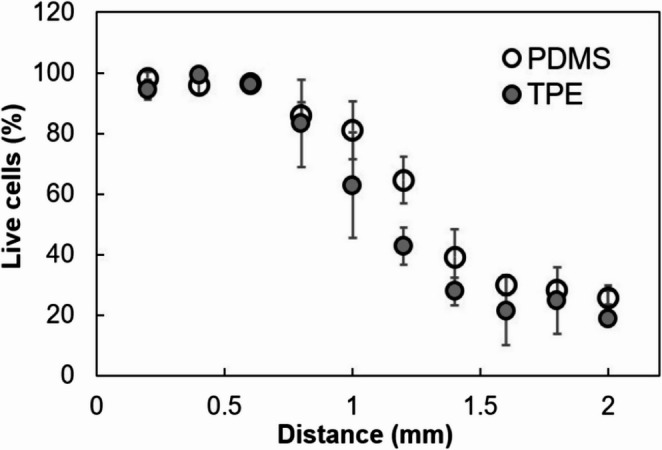
Quantification of live cell percentages at various positions across the chamber for PDMS (*n* = 4) and TPE (*n* = 3) microfluidic devices

Figure 6 shows a comparative graph of live cell distribution across the chamber in the MCGG system for devices fabricated using both TPE and PDMS materials. As expected, in the media-exposed region, the percentage of live cells in both systems remains above 90% and gradually decreases toward the drug-exposed area due to its cytotoxic effects on the cells. The small variation observed in the central region in Fig. 6 may be caused by the converging flow of the two streams. Although slight differences are noted between the two devices there was no statistically significant differences in the overall cell viability across the channel. This indicated that TPE-glass bonded devices perform comparably to PDMS-glass devices in supporting live cells for drug testing using the MCGG system.

## Conclusions

We presented the design and fabrication of TPE-based microfluidic chips capable of generating and controlling concentration gradients as a viable alternative to PDMS devices for cell culture applications. The performance of these chips was validated through both numerical simulations and experimental tests using co-flowing fluorescent dyes. The devices were employed to study the response of cultured human lung fibroblast cells to concentration gradients of FBS. We were able to tailor the concentration gradient profiles within the cell chamber by modifying the chip design parameters, such as microchannel width, microchannel depth, and cell culture chamber dimensions. Additional adjustments to the flow direction and the flow rate enabled fine-tuning of the gradient profiles. We also demonstrated successful culture of human colon adenocarcinoma cells (HT-29) on these chips and evaluated their response to gradients of sepantronimum bromide (YM-155). The results were benchmarked against equivalent PDMS-fabricated chips, illustrating the efficacy of TPE-based devices for drug testing and cell culture applications. The versatility and strong bonding capability of TPE, particularly its ability to conformally bond to other thermoplastic materials(Moon et al. 2021, 2024) makes it a promising material for future pre-clinical applications. Furthermore, our approach addresses key challenges in scalability in microfluidic systems. In general, these advances show that TPE-based microfluidic platforms could make significant contributions to personalized medicine and organ-on-a-chip technologies, delivering precise drug delivery systems and physiologically relevant models.

## Supplementary Information

Below is the link to the electronic supplementary material.

Supplementary Material 1 (DOCX 3.06 MB)

## Data Availability

No datasets were generated or analysed during the current study.
