# Supplementary material for: Thermoplastic elastomer based microfluidic gradient generator for cell culture and drug testing
Source: Biomed Microdevices. 2026 Apr 25;28(2):32. doi: 10.1007/s10544-026-00816-y (PMC13110213; doi:10.1007/s10544-026-00816-y)
Supplement: Supplementary file 1 — Supplementary Material 1 (DOCX 3.06 MB) [file 10544_2026_816_MOESM1_ESM.docx]

**Supporting Information**

Thermoplastic elastomer based microfluidic gradient generator for cell culture and drug testing

Kebin Li^1,3†^, Byeong-Ui Moon^1,3†^, Liviu Clime^1,3^, Ljuboje Lukic^1^, Keith Morton^1,3^, Lidija Malic^1,3^, Anu David^2^, Christophe Faure^2^, and Teodor Veres^1,3^

^1^National Research Council, Boucherville, Canada

^2^ CHU Saint-Justine Research Center, University of Montreal, Montreal, Canada

^3^ Centre for Research and Applications in Fluidic Technologies (CRAFT), University of Toronto, Toronto, Canada

† These authors contributed equally to the manuscript

^*^Email address: [Kebin.li@cnrc-nrc.gc.ca](mailto:Kebin.li@cnrc-nrc.gc.ca)

**Table S1**. Comparing table of some physical properties of TPE and PDMS

**Figure S1.** Concentration fields for two different design MCGGs chips modeled by using COMSOL (version 3.4) at a flow rate of 0.1µL/min. (a) in design 1 (same width for the communication channels, with two outlets, (b) in design version 2 (varied channel width for communication channels), outlet close to the narrow channels, (c) in design version 2 (varied channels width for communication channels), outlet closer to wider channels.

**Figure S2.** Profile of concentration gradients for both Rh-B and FITC-DEX at different locations of the chamber in design 2 with varied width of the communication channels. (a) at a flow rate of 1µL/min. (b) at a flow rate of 100 nL/min (c) at a flow rate of 50 nL/min (flow direction: narrow to wide).


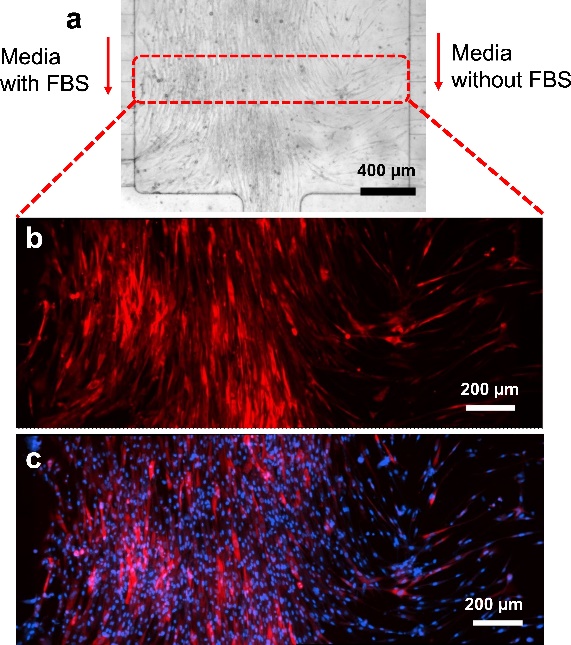


**Figure S3.** Microscopy images of fibroblast culture on the MCGG device. The cells were cultured in a co-flow stream of media with 10% FBS (left channel) and without FBS (right channel) for 2 days at a flow rate of 100 nL/min (a). One section of the image (dotted line box in (a)) shows red fluorescence protein-expressed fibroblasts (b) and its merged image with Hoechst staining (c).


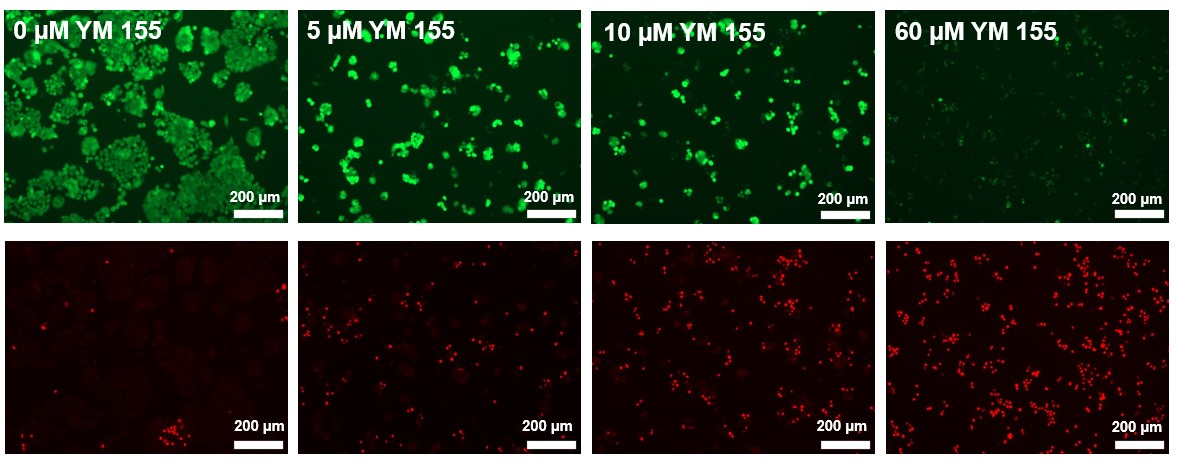


**Figure S4.** YM-155 drug response in HT-29 culture in a 96-well plate. After 2 days of drug exposure, a live/dead assay was performed. Top panel and bottom panels show representative microscopy images of live and dead cells, respectively, at concentrations of 0, 5, 10, and 60 µM.


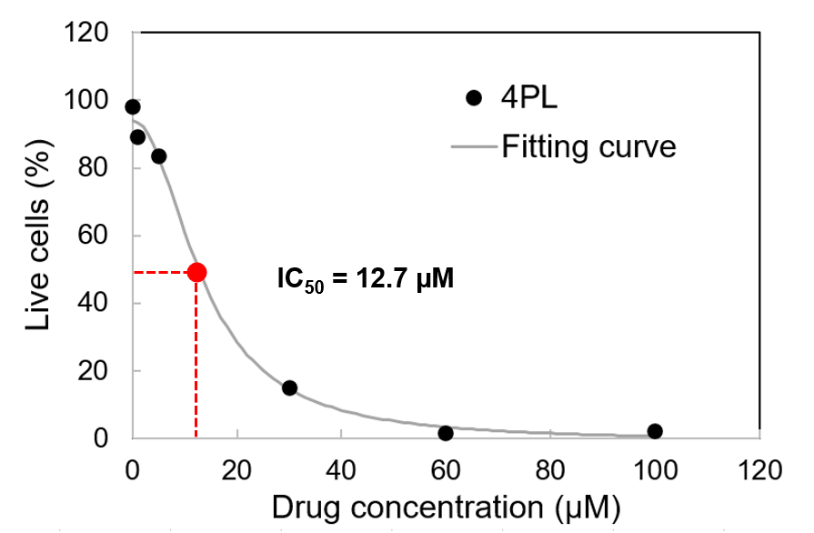


**Figure S5.** Fitting dose-response curve of the HT-29 treated with the YM-155 inhibitor. The IC_50_ was determined using four-parameter logistic (4PL) model: y = Bottom + $\frac{Top-Bottom}{{1+(x/IC50)}^{\mathrm{Hillslope}}}$ , where Top represents the maximum viability, Bottom represents the minimum viability, and Hillslope represents the steepness of the curve. The calculated IC₅₀ value is 12.7 µM.


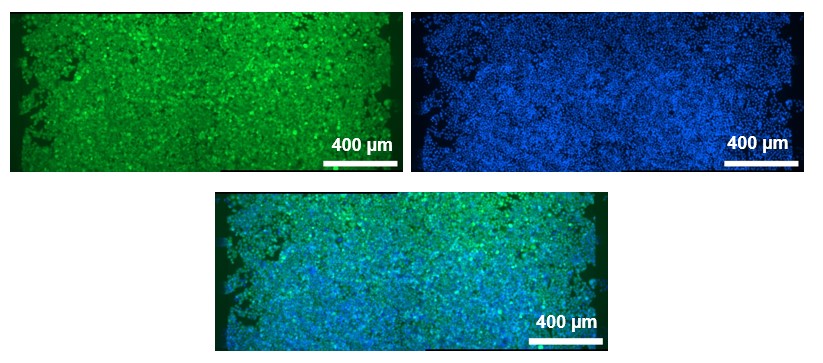


**Figure S6.** HT-29 cell culture under static flow conditions without drug exposure used as a control. The images show live cells (green, top left), Hoechst-stained nuclei (blue, top right), and merged images (bottom).

**Incompressible Navier-Stokes (NS) equations:**

$\text{ρ}\frac{\text{∂}\text{u}}{\text{∂t}}\text{+}\text{ρ}\text{u}\text{∙}\text{∇}\text{u}\text{=}\text{∇}\text{∙}\left[ \text{-}\text{pI}\text{+}\text{}\left( \text{∇}\text{u}\text{+}\left( \text{∇}\text{u} \right)^{\text{T}} \right) \right]\text{+F}$ (1)

$\text{∇}\text{∙}\text{u}\text{=0}$ (2)

Where ρ is the medium’s mass density, **u** denotes the velocity vector, p stands for the pressure, **I** is the identity matrix, η is the dynamic viscosity, F signifies the vector of external body forces (not accounted for here), *t* refers to the time, and ∇ is the standard vector differential operator. The simulation applied the physical properties of water to the fluids involved, with a density of ρ=1000 kg/m^-3^ and a dynamic viscosity of η=10^-3^ kg m^-1^ s^-1^. A diffusion coefficient of D=4×10^-10^ m^2^s^-1^ (for Rh-B) was used for the fluids in the simulations.

**Mass transport equation as implemented in the Chemical Engineering Module:**

$\frac{\partial c}{\partial t}+\nabla\cdot\left( -D\nabla c \right)=-\mathbf{u}\cdot\nabla c$ (3)

Here *c* represents the concentration of a solute, *D* is the diffusion coefficient, and **u** denotes the local velocity vector.

**Initial and boundary conditions are listed below:**

$u(t=0)=0$ (4)

$\boldsymbol{u}=-U_{0}\boldsymbol{n}$ (5)

$\boldsymbol{n}\cdot\boldsymbol{u}=0,[-pI+(\nabla\boldsymbol{u}+{(\nabla\boldsymbol{u})}^{T})]\boldsymbol{n=0,}p=p_{0}$ (6)

$\boldsymbol{c=}\boldsymbol{c}_{\boldsymbol{0}}$ (7)

$n\cdot\left( -D\nabla c \right)=0$ (8)

$n\cdot N=0;N=-D\nabla c+c\mathbf{u}$ (9)

The inlet boundary condition for the flow equations was imposed as a constant flow rate with normal inflow velocity (Eq. (5)), while the outlet was set to constant pressure with stress free boundary condition, restricting the solver to maintain the pressure at a given value p_0_=0 (Eq. (6)). No-slip boundary conditions, indicating zero velocity **u**=0, were applied to all the internal walls. For the mass transport model, a solute concentration c_0_=0.2 mM was considered along with inflow conditions (Eq. (7)), whereas the outflow was configured to ignore diffusive transport across the outlet border (Eq. (8)), and the insulation or symmetry was applied to all internal and external walls (Eq. (9), where N stands for the inward flux)

**References:**

1. E. Roy, M. Geissler, J. Galas and T. Veres, Microfluid and Nanofluid **11**:235–244 (2011), DOI 10.1007/s10404-011-0789-2.
2. E. Roy, J. Galas and T. Veres, Lab Chip **11**, 3193 (2011)
3. A. H. McMillan, J. Mora-Macías, J. Teyssandier, R. Thür, E. Roy, I. Ochoa, S. De Feyter, I. F. J. Vankelecom, M. B. J. Roeffaers and S. C. Lesher-Pérez, *Nano Select* **2**, 1385–1402 (2021).
4. K. Domansky, J. D. Sliz, N. Wen, C. Hinojosa, G. Thompson, J. P. Fraser, T. Hamkins-Indik, G. A. Hamilton, D. Levner and D. E. Ingber, *Microfluid and Nanofluid* **21**, 107 (2017).
5. Byeong-Ui Moon, K. Morton, K. Li, C. Miville-Godin and T. Veres, Processes **9**, 54 (2019). <https://doi.org/10.3390/pr9010054>
6. Byeong-Ui Moon, K. Li, L. Malic, K. Morton, H. Shao, L. Banh, S.Viswanathan, E W. K. Young and T. Veres, Lab Chip **24**, 4948–4961 (2024).
